# Wide-field quantitative micro-elastography of freshly excised human prostate

**DOI:** 10.1364/BOE.563310

**Published:** 2025-07-03

**Authors:** Szymon Tamborski, Marta K. Skrok, Matt S. Hepburn, Mateusz Maniewski, Marek Zdrenka, Adam Kowalewski, Łukasz Szylberg, Brendan F. Kennedy

**Affiliations:** 1 Institute of Physics, Faculty of Physics, Astronomy and Informatics, Nicolaus Copernicus University in Toruń, 5 Grudziądzka St., 87-100 Toruń, Poland; 2 Department of Electrical, Electronic and Computer Engineering, School of Engineering, The University of Western Australia, 35 Stirling Highway, Perth, Western Australia 6009, Australia; 3 BRITElab, Harry Perkins Institute of Medical Research, QEII Medical Centre Nedlands and Centre for Medical Research, The University of Western Australia, Perth, Western Australia 6009, Australia; 4 Department of Obstetrics, Gynaecology and Oncology, Chair of Pathomorphology and Clinical Placentology, Collegium Medicum in Bydgoszcz, Nicolaus Copernicus University in Toruń, 75 Ujejskiego St., Bydgoszcz 85-168, Poland; 5 Department of Tumor Pathology and Pathomorphology, Oncology Centre Prof. Franciszek Łukaszczyk Memorial Hospital, 2 Romanowskiej St., Bydgoszcz 85-796, Poland; 6 Faculty of Medicine, Bydgoszcz University of Science and Technology, Prof. S. Kaliskiego Avenue 7, 85-796 Bydgoszcz, Poland

## Abstract

Prostate cancer is a significant global health issue. To advance diagnostic and treatment procedures, such as image-guided needle biopsy, optical imaging methods offering high spatial resolution have been proposed. Optical coherence tomography (OCT) allows for detailed visualization of prostate microarchitecture in three dimensions; however, it does not reliably differentiate tumors from surrounding healthy tissue. In this study, we use wide-field quantitative micro-elastography (QME) to image the micro-scale mechanical properties of freshly excised human prostate to provide additional contrast of tumor, which is known to exhibit increased stiffness. In our approach, we generate volumetric OCT, strain, and elasticity images over a field-of-view (*x* × *y* × *z*) of 46 × 46 × 1 mm^3^. We validate the contrast obtained in our images through co-registration with standard histology images.

## 1. Introduction

Globally, prostate cancer is the second most commonly diagnosed cancer in men with approximately 1.5 million new cases recorded and ∼400,000 deaths each year [1]. Early and accurate diagnosis is critical to reduce both the mortality rate and complications following surgery, such as infertility, erectile dysfunction, and urinary incontinence [2]. Medical imaging plays an important role in prostate cancer diagnosis. Currently, a gold standard in clinical practice is transrectal ultrasound (TRUS), which is used to assess pathological changes in prostate structure and as a monitoring tool for biopsy guidance. However, the effectiveness of standard TRUS-guided biopsy is limited by its low spatial resolution (∼200 µm) and low contrast, with false-negative results exceeding 30% until recently [3]. Recently, in micro-ultrasonography, using high-frequency ultrasound waves, resolution as high as 70 µm was achieved, resulting in a significant improvement in the accuracy of cancer detection in biopsies [4]. Furthermore, higher spatial resolution is also essential in guiding focal tumor removal techniques, such as ablation using high-intensity focused ultrasound (HIFU) [5]. Another imaging modality that has been applied to prostate cancer is magnetic resonance imaging (MRI), which showed high accuracy in the identification of cancerous regions [6]. The approach, including multi-parameter MRI fusion with real-time ultrasonography, is considered one of the most accurate imaging methods for biopsy guidance [7]. In recent years, many new MRI techniques for prostate imaging have been proposed to improve both cancer detection and therapeutic interventions [8], including hybrid multidimensional MRI [9], magnetic resonance fingerprinting [10], and restricted spectrum imaging [11]. To address the problem of insufficient resolution, optical imaging modalities have been also proposed for use in biopsy guidance, as these techniques have higher spatial resolution and are often compatible with compact imaging probes. To date, most studies have focused on proof-of-concept experiments, mainly on *ex vivo* biopsy cores. These studies have demonstrated the feasibility of differentiating tumor using techniques including photoacoustic tomography [12], multiphoton microscopy [13], Raman spectroscopy [14], and light sheet microscopy [15]. However, these techniques either lack volumetric imaging capabilities, provide limited field-of-view (FOV) or require optical clearing of the tissue. Optical coherence tomography (OCT) has also shown promise in imaging prostate tissue based on intrinsic contrast from optical backscattering [16–22]. OCT offers a unique combination of volumetric imaging capabilities with micrometer–scale resolution to a depth of ∼1 mm in scattering tissue at high acquisition speeds that provide real-time tissue visualization [16]. It does not require staining of the imaged sample as the signal carriers are the photons backscattered from the imaged volume and thereby uses the object’s intrinsic optical contrast. Several preliminary studies using time-domain OCT demonstrated the potential of OCT imaging of the prostate [17,18]. However, the relatively low spatial resolution and sensitivity of these OCT systems made it difficult to visualize micro-architecture and to differentiate tissue types within the prostate. Gardecki *et al*. demonstrated higher spatial resolution using optical coherence microscopy (OCM), providing an axial resolution of 1 µm and lateral resolution of 2 µm. Using this approach, the potential to provide detailed images of prostate features, including neoplastic changes, was demonstrated in fresh biopsy specimens [19]. However, this high resolution was obtained at the cost of a small lateral imaging FOV (1 × 1 mm^2^) and a relatively low imaging depth (∼300 µm), limiting the potential for clinical applications, which often require inspection of regions deep within the organ. One proposed solution to the limited imaging depth of both OCT and OCM is the OCT needle probe, where the sample arm of the OCT system is miniaturized and placed within a standard biopsy needle to allow investigation of prostate tissue to depths of centimeters [20]. An *in vivo* feasibility study was performed using an OCT needle probe, where validation of OCT contrast was obtained through co-registration with histology performed on corresponding biopsies taken from the regions scanned with the probe [21]. The main challenge of this approach remains the limited accuracy of co-registration. In an *ex vivo* study on a benchtop OCT system, our group recently reported on the capability of OCT to identify various structures within entire cross-sections of fresh prostatectomies, including nerves, blood vessels, and cancer at different stages [22]. However, these studies have shown that, in some cases, OCT exhibits limited contrast between cancer and surrounding benign tissue.

Functional extensions of OCT have the potential to improve the visualization of prostate cancer. For example, Swaan *et al*. [23] demonstrated improved contrast using OCT attenuation imaging, at the expense of relatively low axial resolution. Another possibility is to generate contrast based on the increased stiffness of prostate cancer. On the macro-scale, modifications to the mechanical properties of prostate cancer form the basis for digital rectal examination (DRE), where the clinician utilizes manual palpation to provide an assessment of the prostate [24]. Despite high subjectivity and access to only the back wall of the prostate, it remains a main screening examination for the detection of prostate cancer. Given this established link between tissue stiffness and prostate cancer on the macro-scale and, also, given that ultrasound elastography has been demonstrated to identify prostate cancer [25], there is distinct potential to use optical coherence elastography (OCE) to provide improved contrast in the identification of prostate cancer on the micro-scale. OCE is a functional extension of OCT that measures tissue deformation in response to an applied mechanical load [26,27]. Using a mechanical model based on solid mechanics, a mechanical property of the tissue, most commonly elasticity, is estimated from the measured deformation. OCE has demonstrated high diagnostic accuracy (>90%) in the detection of cancer in excised breast specimens [28,29] and has also been proposed for *in vivo* imaging of cancer in the breast surgical cavity [30]. In addition, several studies have demonstrated the potential of OCE to identify prostate cancer. Li *et al*. [31] presented a study of 120 biopsy-derived prostate specimens using vibrational OCE and showed statistical differences between healthy tissue and cancerous tissue, providing high sensitivity (98%) and specificity (91%) in cancer detection. The same group also reported a study in which OCE was correlated with second-harmonic generation imaging to determine the orientation of collagen fibers as a proposed biomarker for neoplastic lesions [32]. However, in both studies, prior to imaging the specimens were fixed in formalin, which is known to increase elasticity by up to 120% [33]. Furthermore, the change in elasticity caused by fixation is dependent on the tissue type. As a result, the clinical relevance of these previous studies is unclear. In addition, whilst the elasticity of prostate biopsies was quantified, the capability to map elasticity at a spatial resolution high enough to distinguish key prostate features was not shown, further limiting the potential for clinical use.

Our approach applies a compression OCE technique, quantitative micro-elastography (QME) [34], to image freshly excised prostate tissue. QME provides information on the spatially resolved values of elasticity of the tissue expressed in kilopascals, i.e., the same as ultrasound elastography, but with a spatial resolution almost two orders of magnitude higher. As such, it offers an additional source of contrast that has the potential to provide precise tumor localization, which is beyond the reach of more established optical imaging methods, such as OCT. High resolution and high sensitivity provide the potential to distinguish between particular Gleason patterns, and thus avoid unnecessary surgical interventions, similar to ultrasound elastography. Specifically, QME has the potential to distinguish between cancer and benign prostatic hyperplasia, which cannot be reliably made with DRE. Such distinction is challenging using alternative optical methods, including OCT. An important advantage of QME is also compatibility with imaging probes that will be required for eventual clinical translation in for example biopsy guidance [30].

In this paper, we performed QME on 18 sections dissected from 11 freshly excised human prostates. Importantly, instead of focusing on biopsy cores, in this proof-of-principle study, we characterize the micro-scale elasticity of large regions of the prostate by imaging entire cross-sections of fresh tissue immediately following prostatectomy. This was achieved by acquiring multiple, partially overlapped volumes and mosaicking them to extend the lateral FOV to 46 × 46 mm^2^, similar to an approach previously used on breast specimens [35]. Using this approach, we obtained three-dimensional (3-D) images of tissue micro-structure using OCT, as well as 3-D images of strain and elasticity using OCE. Validation of the contrast obtained in OCT images and elastograms was provided by co-registering the images with hematoxylin and eosin (H&E) stained wide-field histology images of the same prostate slice.

Our results indicate that regions of healthy tissue often present with high strain heterogeneity, largely resulting from the high density of prostate glands surrounded by stroma. Conversely, in regions of tumor, strain typically presents as more homogeneous, resulting from the high density of tumor cells, which causes the tissue to deform more uniformly. In elasticity images, tumor typically presents with higher elasticity than the surrounding healthy tissue. In general, our results indicate that both strain and elasticity images provide significantly higher contrast within the prostate and have the potential to identify tumors with greater accuracy than OCT alone.

## 2. Methods

### 2.1. QME imaging setup

**Fig. 1.**
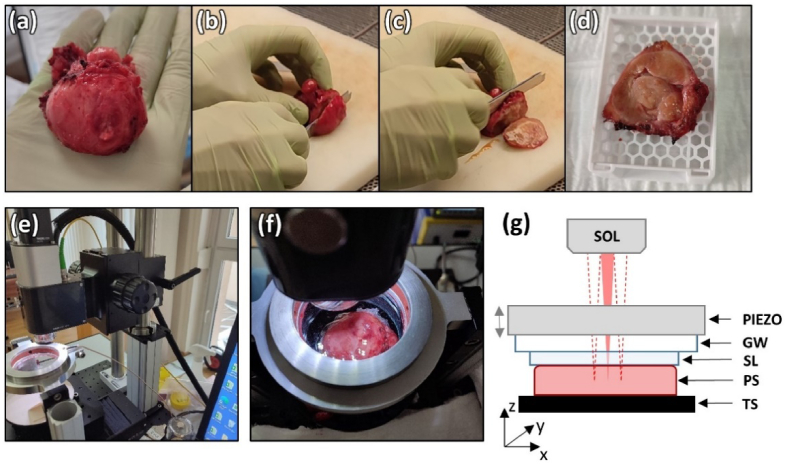
(a) A whole fresh prostate after radical prostatectomy. (b) Dissection of the external part of the peripheral zone including the apex. (c) The dissection of the slice to be imaged in the axial plane. (d) Prostate slice to be imaged in the macro-cassette. (e) QME imaging system on-site at the Oncology Centre. (f) The prostate sample covered with a silicone layer during scanning. (g) Illustration of the sample arrangement for QME imaging. SOL – scanning objective lens, PIEZO – ring piezo actuator, GW – glass window, SL – silicone layer, PS – prostate sample, TS – three-axis translation stage.

The QME system used in this study has been described in detail previously [35]. In brief, it is based on a Telesto II OCT system (TEL220C1, Thorlabs, USA). It features a broadband light source with a 200 nm bandwidth centered at 1300 nm, providing an axial resolution of 5.5 µm in air. The scanning head combines a pair of galvanometric scanners with a scanning lens, providing a lateral resolution of 13 µm. The spectrometer includes a line-scan camera with 2048 pixels, providing an axial imaging range of 3.5 mm in air. Typically, attenuation of the OCT intensity in dense tissues, such as the prostate, limits the imaging depth to ∼1 mm. The beam is incident from above the sample through the imaging window (6 mm thickness, fused silica, Edmund Optics). The telecentricity of the imaging optics enables common-path acquisition with low signal drop-off at the edges of the lateral FOV. Implementation of a common-path configuration ensures high phase sensitivity despite the imaging system being mounted on a standard laboratory bench (Fig. 1(e)). The reference reflection is provided by the interface between the imaging window and the top surface of the compliant silicone layer (see Fig. 1(g)) placed between the imaging window and the sample. The displacement sensitivity of the system was 1.44 nm measured at an OCT signal-to-noise ratio of 40 dB. An additional visible light channel, which is combined with the OCT path using a dichroic mirror, facilitates sample positioning using a motorized 3-axis stage. Images obtained in this channel are also useful in co-registration of the OCE images with histology.

The parameters of the scanning protocol were based on a previous study on breast tissue [35]. For a single volume, the QME scanning pattern comprised 808 A-scans acquired along the *x*-axis per B-scan and 2424 B-scan pairs acquired along the *y*-axis over a lateral FOV of 16 × 16 mm^2^. For each location on the *y*-axis, B-scans were acquired in pairs for two levels of mechanical compression of the sample. The details of this protocol are described in the *QME scanning* subsection. For OCT imaging, B-scans acquired at every three adjacent locations in the *y*-axis were averaged to reduce optical noise, resulting in isotropic sampling in both lateral dimensions (808 × 808 pixels). The spectrometer acquired spectra with an exposure time of 12 µs and a line period of 16 µs. The B-scan rate was ∼68.8 Hz. To acquire data over an entire prostate slice in a wide-field mode, we used a previously established protocol [35], which involved mosaicking a 3 × 3 grid of volumes with an overlap region of 1 mm. This was achieved by mounting the specimen on motorized translation stages. Data acquisition was controlled by custom software. A single volume was acquired in ∼70.4 seconds, resulting in a total acquisition time for wide-field images of ∼12 minutes. This procedure resulted in wide-field OCT, strain, and elasticity images over a FOV of 46 × 46 × 2.6 mm^3^ (*x* × *y* × *z*). For the purpose of co-registration, we used *en face* projections obtained for a particular depth within the sample.

### 2.2. QME scanning

QME is a compression OCE technique in which the local response of the tissue to applied stress is measured. In our setup, the stress was applied using a transparent glass window mounted in an aperture of the ring piezoelectric actuator. The window was placed within the working distance of the objective lens of an OCT system [34]. The axis of movement of the actuator was aligned with the optical axis of the scanning objective lens (Fig. 1(g)). Before imaging, the preload was applied to the sample by squeezing it against the imaging window using the translational stage in the *z*-axis (Fig. 1(f)). Thus, applied stress at the level of several kilopascals resulted in a strain of 15-20%. The actuator working with the stroke of ∼10 µm enabled the uniform application of additional micro-scale stress to the surface of the sample. The measurement was carried out by consecutively acquiring two B-scans at two different levels of compression at each *y*-location of the scan pattern. This involved synchronization of the piezo actuator driven by a rectangular wave-form and B-scan acquisition. After standard OCT processing steps involving Fourier transformation of the originally acquired intereference spectra, complex B-scans were obtained. In the next step, the phase difference between the two B-scans within a pair acquired in each position along the *y*-axis was calculated. The phase differences obtained for three adjacent B-scans pairs were averaged to alleviate the deleterious effects of optical noise and OCT speckle and resulted in uniform sampling in the *x*- and *y*-axes. The resulting QME scan volume comprises 808 × 808 × 1024 (*x* × *y* × *z*) voxels with a voxel size of 20 × 20 × 2.6 µm^3^. Resultant phase-difference B-scans were used to calculate the local displacement of the tissue in the axial direction using information about the light spectrum and assuming average refractive index of the tissue (*n* = 1.33). Local strain was then estimated from the displacement map using one-dimensional weighted least-squares linear regression over a fitting range of 100 µm in *z* [36]. Estimation of elasticity requires knowledge of the applied stress, which, in QME, is measured by first estimating the strain in a compliant transparent silicone layer (made with Elastosil P7676, Wacker Chemie AG, Germany, hardness: 15 in Shore 00 scale, tensile strength 0.6 N/mm^2^)(Fig. 1(g)). Using the pre-calibrated stress-strain response of the layer and its thickness measured with micrometer-scale resolution using OCT (with an average thickness of ∼0.5 mm), the stress at each lateral location at the sample surface was calculated [34]. Assuming that the stress in tissue is uniform and uniaxial and having the spatially resolved maps of the strain, the local elasticity, defined as the ratio of stress over strain was calculated. To minimize the influence of friction at the glass-silicone interface, the layer was lubricated with silicone oil before being brought into contact with the window [37]. Post-processing of the data using custom-developed software provides volumetric local strain and elasticity. The wide-field *en face* strain and elasticity projections with the lateral FOV of 46 × 46 mm^2^ were obtained by stitching the projections from all the volumes at a particular depth in the same way the wide-field OCT *en face* projections were generated. For comprehensive visualization, the strain and elasticity images were overlaid on the OCT image.

### 2.3. Clinical scanning

All imaging was performed at the Oncology Centre in Bydgoszcz, Poland, and the study received approval from the Bioethics Committee of Nicolaus Copernicus University. Eighteen specimens from 11 freshly excised prostates obtained through radical prostatectomy were scanned. The average age of the patients was 69.9 ± 6.3 (mean ± standard deviation) years. All the patients suffered from prostatic lobular adenocarcinoma confirmed by the standard post-prostatectomy histology procedure with an average Gleason score of 7.7 ± 0.9 (mean ± standard deviation). In each case, a slice with a thickness of ∼5 mm was taken in the axial plane, approximately 1 cm below the apex tip, by a qualified pathologist within 30 minutes of excision (see Figs. 1(a)–(d)). The slice was then immediately transported to the QME imaging system. In cases of prostate enlargement, where the slice exceeded the maximum scanning area of the wide-field QME system, the slice was bisected along the sagittal plane, and each section was scanned separately (N = 4). In each case, the sample was irrigated with saline solution, before being covered with a silicone layer. Following QME acquisition, the sample was removed from the sample stage, irrigated again, and placed in a macro-cassette. The standard protocol was then used to prepare hematoxylin and eosin (H&E) stained histology images. In each case, histology images were generated from the area scanned by QME at a depth of ∼100 µm below the tissue surface. The use of macro-cassettes allowed for histology images as large as 50 × 64 mm^2^, closely matching the QME wide-field lateral scanning area. This enabled the acquisition of a single scan of the entire specimen, greatly facilitating subsequent co-registration with histology. Within the collection of scanned specimens, Gleason pattern 3 was observed in 10 specimens, Gleason 4 pattern was found in 12 specimens, and Gleason 5 was identified in 4 specimens. In one specimen, no neoplastic changes were observed.

### 2.4. Gleason grading

Throughout this manuscript, we refer to the Gleason grading system. This is the gold-standard method for describing neoplastic changes in prostate microarchitecture, used for both the diagnosis and prognosis of prostate cancer [38]. It recognizes five grades of prostate neoplastic patterns, ranging from 1 to 5 with increasing severity. Each grade is defined by characteristic features in the prostate micro-architecture assessed by histology. In prostates affected by cancer, different patterns can be present, covering varying percentages of the sample’s area. In medical practice, the Gleason score is used as the diagnostic parameter of the advancement of the tumor development. The Gleason score is defined as the sum of the grades of the dominant pattern and next-most frequent pattern present in the investigated prostate specimens. Importantly, elevated stiffness measured with ultrasound elastography shows a correlation with increased Gleason score [39].

### 2.5. Co-registration between QME and histology

Co-registration between QME and histology was achieved through a detailed comparative inspection of the histology image by a qualified pathologist with the corresponding OCT, strain, elasticity images, and visible light camera images. Characteristic features were identified in the images, which enabled them to be oriented relative to each other to visually maximize their similarity. Characteristic structures in the images, such as stromal bands or the location of residual periprostatic tissue, were identified to assess possible changes in the geometry of the sample that could occur in the multi-step histology sample preparation process. Annotations provided by qualified pathologists were used for the interpretation of the OCT, strain, and elasticity images.

## 3. Results

**Fig. 2.**
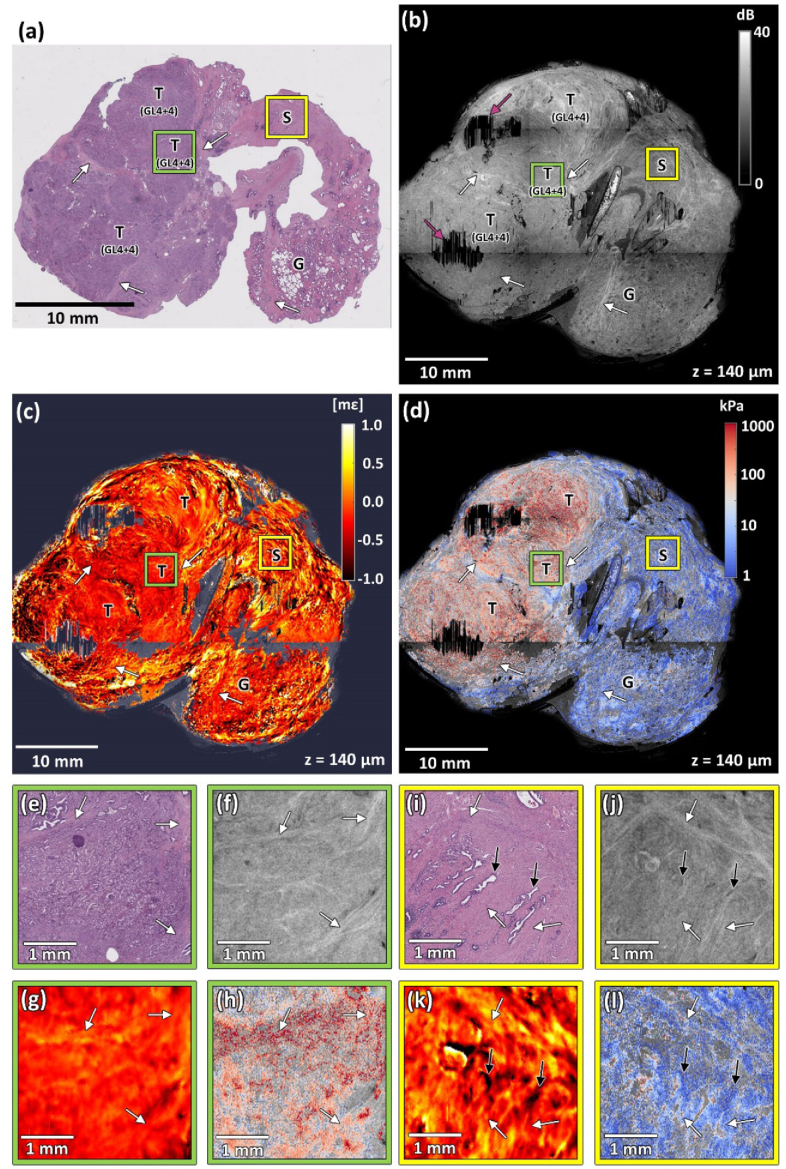
Co-registered images of Specimen 1 with advanced adenocarcinoma with Gleason score 8 (4 + 4). (a) Histology image. (b) OCT image. (c) Strain image. (d) Elasticity image. (e)–(h) Magnified region containing tumor (Gleason score 8 (4 + 4)) marked with green squares in panels (a)–(d). (i)–(l) Magnified region containing mostly normal stroma marked with yellow squares in panels (a)–(d). T – tumor, G – glands, S – stroma, GL(4 + 4) – Gleason score 8 (4 + 4). White arrows – characteristic fibromuscular bands. Black arrows – singular normal glands. Purple arrows – artifacts caused by the erroneous behavior of the layer detection algorithm in areas of high surface roughness. OCT, strain, and elasticity images are taken from a depth of ∼140 µm below the tissue surface.

**Fig. 3.**
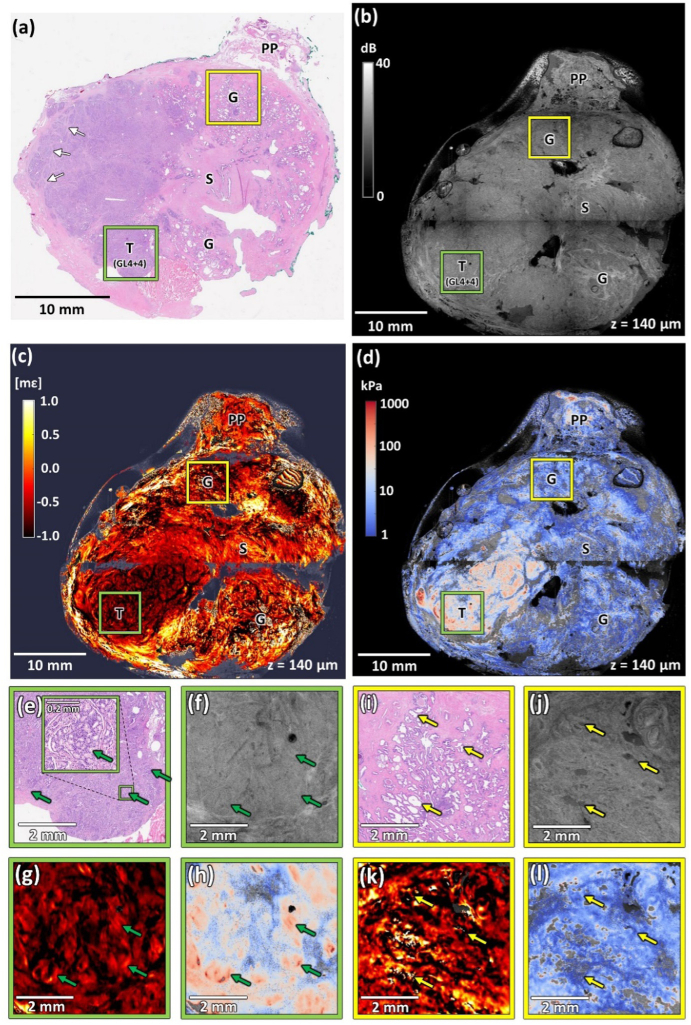
Co-registered images of Specimen 2 with discrete tumor clusters. (a) Histology image. (b) OCT image. (c) Strain image. (d) Elasticity image. (e)–(h) Magnified region containing tumor (Gleason score 8 (4 + 4)) marked with green squares in panels (a)–(d). (i)–(l) Magnified region containing healthy glands marked with yellow squares in panels (a)–(d). T – tumor, G – glands, S – stroma, PP – residual periprostatic tissue, GL(4 + 4) – Gleason score 8 (4 + 4). White arrows – characteristic fibromuscular bands. Yellow arrows – examples of healthy glands. Green arrows – tumor clusters. OCT, strain, and elasticity images are taken from a depth of ∼140 µm.

**Fig. 4.**
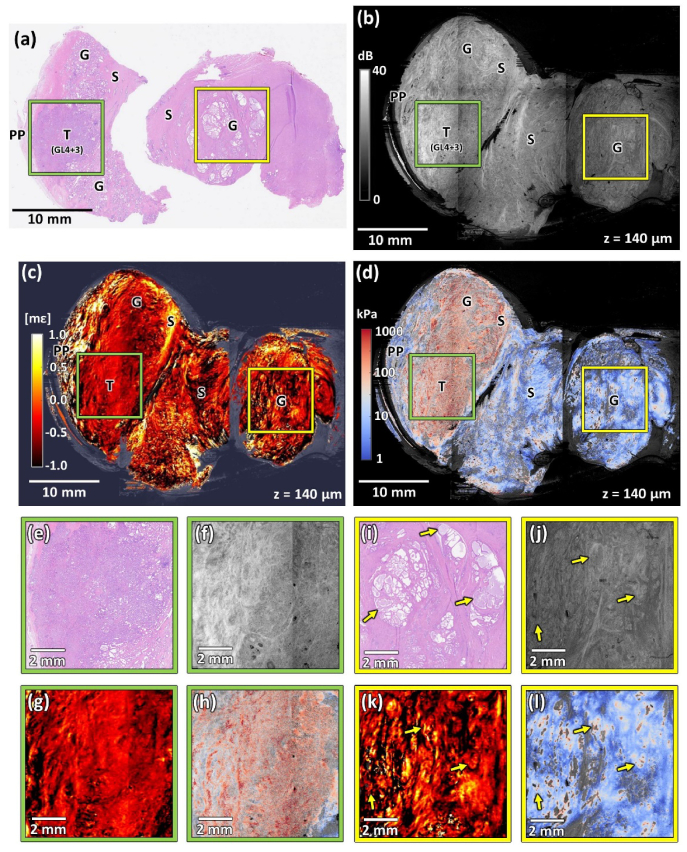
Co-registered images of Specimen 3 with cancerous and hyperplastic changes. (a) Histology image. (b) OCT image. (c) Strain image. (d) Elasticity image. (e)–(h) Magnified area containing tumor (Gleason score 7 (4 + 3)) marked with the green squares in panels (a)–(d). (i)–(l) Magnified area containing healthy glands marked with the yellow squares in panels (a)–(d). T – tumor, G – glands, S – stroma, PP – periprostatic tissue, GL(4 + 3) – Gleason score 7 (4 + 3). Yellow arrows – enlarged glands filled with residual prostatic fluid. OCT, strain, and elasticity images are taken from a depth of ∼140 µm.

**Fig. 5.**
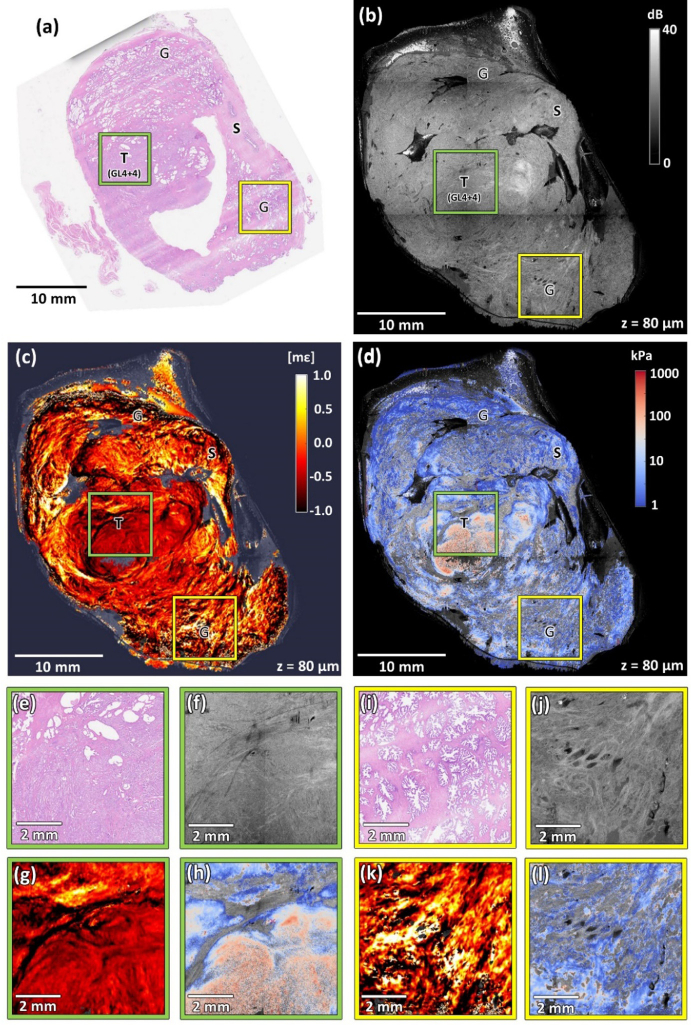
Co-registered images of Specimen 4 with Gleason score 8 (4 + 4) tumor and extensive regions of normal glands. (a) Histology image. (b) OCT image. (c) Strain image. (d) Elasticity image. (e)–(h) Magnified area containing tumor (Gleason score 8 (4 + 4)) marked with the green squares in panels (a)–(d). (i)–(l) Magnified area containing healthy glands marked with the yellow squares in panels (a)–(d). T – tumor, G – glands. S – stroma, GL(4 + 4) – Gleason score 8 (4 + 4). OCT, strain, and elasticity images are taken from a depth of ∼80 µm.

**Fig. 6.**
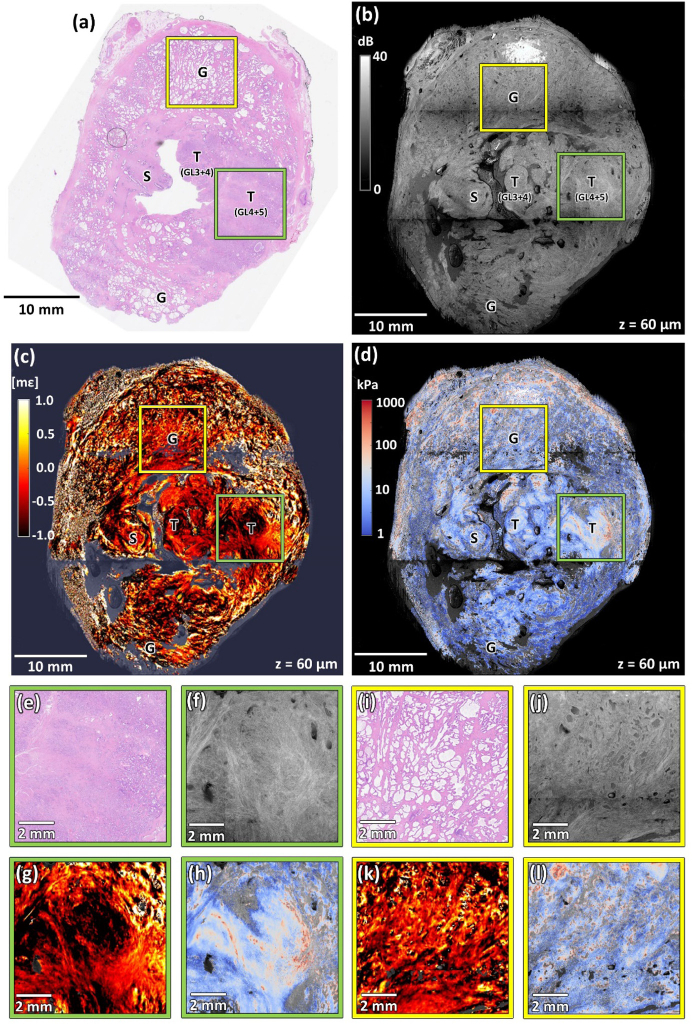
Co-registered images of Specimen 5 with tumor regions of varying Gleason grades from 3 to 5. (a) Histology image. (b) OCT image. (c) Strain image. (d) Elasticity image. (e)–(h) Magnified area containing tumor (Gleason score 9 (4 + 5)) marked with the green squares in panels (a)–(d). (i)–(l) Magnified area containing healthy glands marked with the yellow squares in panels (a)–(d). T – tumor, G – glands, S – stroma, GL(4 + 5) – Gleason score 9 (4 + 5), GL(3 + 4) – Gleason score 7 (3 + 4). OCT, strain, and elasticity images are taken from a depth of ∼60 µm.

In Figs. 2–6, we present wide-field OCT, strain, and elasticity images of slices from the freshly excised prostate co-registered with histology. In OCT images, the contrast is provided by the OCT signal intensity presented in a logarithmic scale. In the strain images, the contrast is provided by the values of strain expressed in dimensionless units (mε), whose negative values depict local compression, and positive values depict tension. The strain values are overlaid on the OCT images. In the elasticity images, the calculated elasticity values expressed in kilopascals (kPa) are also overlaid on the OCT images. We note that the standard convention in mechanical engineering is applied, where both compressive stress and compressive strain are designated as negative. Analogously, positive stress and positive strain indicate tension. Therefore, according to the model of tissue deformation used in QME, which assumes that the stress in tissue is uniform and uniaxial as a response to unidirectional compressive load, both stress and strain are expected to be negative. This scenario results in positive elasticity. However, in compression OCE factors such as surface roughness and mechanical coupling of features within the sample can give rise to unexpected tissue deformation, resulting in positive stress and positive strain, i.e., stress and strain acting in the direction opposite to the applied load. This effect has been described in detail in previous publications [40,41]. If stress and strain have opposite signs, negative elasticity is calculated in QME. As this is not physically meaningful, we remove these regions from elastograms.

Figure 2 shows images of a prostate slice with extensive adenocarcinoma (Specimen 1). The figure comprises co-registered wide-field images of histology (Fig. 2(a)), *en face* OCT (Fig. 2(b)), strain (Fig. 2(c)), and elasticity (Fig. 2(d)), at a depth of ∼140 µm below the sample surface. Figures 2(i)–(l) present a magnified region composed mostly of normal prostate stroma, indicated by the yellow squares in Figs. 2(a)–(d). In general, this type of connective stroma is comprised of fibroblasts, myofibroblasts, smooth muscle cells, vascular endothelial cells, nerve cells, and inflammatory cells [42]. In histology, for example, the region highlighted in Fig. 2(i), presents as a homogeneous area of mainly benign structures, with the cells forming distinguishable longitudinal bands marked with white arrows. In Fig. 2(i), singular glands are visible as empty spaces (indicated with black arrows). Figures 2(e)–(h) present a magnified region containing a developed tumor indicated by the green squares in Figs. 2(a)–(d). In the histology image (Fig. 2(a)), the tumor was graded by a pathologist with a Gleason score 8 (4 + 4) and presents as a region with high nuclei density and cribriform, shrunken, merged glands. In Fig. 2(a), also a region containing large, uninvolved glands of different shapes and sizes is located in the bottom right region of the slice.

The OCT image provides visualization of characteristic features of the prostate architecture. For example, larger glands in Fig. 2(b) appear as darker spots with well-defined borders, contrasting with the brighter surrounding stroma. In the magnified region in Fig. 2(j), OCT provides clear differentiation of fibromuscular stroma. Additionally, the narrow, singular glands marked with black arrows appear as darker spots. A magnified OCT image of a region with Gleason grade 4 is shown in Fig. 2(f). In this case, Gleason grade 4 appears as sheets of poorly formed glands. These structures are visible in the histology image (Fig. 2(e)), but in the OCT images, they are visualized as areas of relatively uniform intensity, with only slight variations indicating the microscale architecture of the cancer. This structure is interlaced with bands of well-contrasted supporting stroma, which aligns with our findings presented in a previous paper [22]. However, the contrast between the OCT images of normal stroma and tumor is relatively low (Fig. 2(j) and Fig. 2(f), respectively), making it challenging to reliably differentiate between the two regions.

In the strain image (Fig. 2(c)), the tumor that covers almost the whole left part of the sample appears as relatively homogeneous with low absolute strain (∼0 mε), which corresponds to the typical response of uniform, stiff tissue to uniaxial stress. The magnification of the chosen region of the tumor presented in Fig. 2(g) reveals only minor variations in strain due to the tumor being intermixed with stroma. In contrast, the regions with healthy glands and stroma on the right-hand side of the image are significantly more heterogeneous, showing both positive and negative strain. This heterogeneity is observed in more detail in the magnified image in Fig. 2(k) and is most likely caused by the deformation of compressible glands embedded in the surrounding stroma, analogously to the previously described case of ducts in breast tissue [40].

In the elasticity image in Fig. 2(d) and the corresponding magnified images presented in Fig. 2(l) (normal stroma), and in Fig. 2(h) (tumor), regions of tumor present with high elasticity (>100 kPa, reddish tones on elastogram) compared to relatively low elasticity (<10 kPa, blueish tones on elastogram) in the non-cancerous region to the right. In these images, there are regions where no elasticity information is presented. These regions correspond to locations where positive strain was measured, which, when combined with negative stress, results in negative elasticity, which is not physically meaningful. This issue is described in more detail in the *Discussion* section.

Figure 3 presents an example of a prostate slice (Specimen 2) with regions of advanced Gleason grade 4 with disseminated discrete tumor. In the histology image in Fig. 3(a), extensive regions of cancer are present on the left-hand side of the image. A part of the residual periprostatic tissue adjacent to the prostate, containing adipose tissue, nerves, and blood vessels, is visible in the upper part of the image. The area of the tumor is interspersed with a well-contrasted network of connective tissue fibers, particularly visible in the top left part of the image. Regions of homogeneous, healthy stroma and clusters of healthy glands are located on the right-hand side of Fig. 3(a). Figure 3(i) shows a magnified image of one of these glandular regions indicated in Fig. 3(a) with a yellow square. Examples of uninvolved glands are indicated with yellow arrows.

Figure 3(e) presents a magnified region containing a tumor indicated by the green square in Fig. 3(a). In histopathology, a tumor with a Gleason grade 4 presents various morphologic patterns. In Fig. 2, fused glands without intervening stroma are visible, creating a highly homogeneous condensed structure. In contrast, in Fig. 3(a), the discrete regions of the tumor are separated by stromal bands. These tumor clusters are visible as oval structures and are indicated by the green arrows in the magnified image in Fig. 3(e). An example of such a cluster is also presented in the magnified inset in Fig. 3(e).

In the OCT image in Fig. 3(b), cancerous tissue is visible as a region with relatively homogeneous intensity, with high-intensity bands corresponding to fibromuscular stroma distributed within the cancerous tissue, analogously to Fig. 2(b). In the uninvolved part of the specimen, large glands present with high contrast, as shown in the magnified image in Fig. 3(j). The representative glands are indicated with yellow arrows. The microarchitecture of the glandular tissue is significantly different from the tumor and the differences are clearly seen in Fig. 3(j) and Fig. 3(f). However, in Fig. 3(b), analogously to Specimen 1, the region of normal stroma shows relatively homogeneous characteristics similar to the tumor. It is also noteworthy that the tumor clusters identified in the histology image (Fig. 3(e)) produce low contrast in the OCT image (Fig. 3(f)), and are difficult to differentiate.

In Fig. 3(c), the strain image is presented. The magnified region of healthy glands shown in Fig. 3(k) features much higher strain contrast than in the normal stroma in Specimen 1 (Fig. 2(k)). This high strain heterogeneity, including positive strain measured in many locations, is attributed to the complex deformation of compressible glands embedded in the surrounding stroma. In the area of tissue containing the tumor, the values of strain are relatively low, compared to the corresponding region in Fig. 2(c). A magnification of the cancerous region is shown in Fig. 3(g). The region exhibits more heterogeneity than the tumor present in Specimen 1 (Fig. 2(g)). The strain in Fig. 3(g) is predominantly negative. Discrete regions of the tumor provide a distinct contrast that likely corresponds to the mechanical heterogeneity between cancer clusters and surrounding intermingled stroma. The tumor clusters are stiffer than the adjacent stroma and hence correspond to the locations of low absolute strain (∼0 mε).

The corresponding elasticity image is presented in Fig. 3(d). A magnified region of cancerous tissue indicated by the green square in Fig. 3(d) is presented in Fig. 3(h). A comparison of the elasticity image in Fig. 3(h) and the strain image in Fig. 3(g) shows the correlation between the high elasticity (∼100 kPa) and low absolute strain (∼0 mε) corresponding to the tumor clusters.

It is noteworthy, that the co-registration of the histology (Fig. 3(a)) and elasticity (Fig. 3(d)) does not provide an exact correlation of the regions with the tumor. In the histology image, the tumor covers almost the entire left half of the image, whereas in the elasticity image, high elasticity (>100 kPa) is found only in the bottom left quadrant of the image. This may result from the histology image and the elasticity images being from slightly different planes or it could indicate that in some instances prostate cancer does not result in stiffening of the tissue.This ambiguity is described in more detail in the *Discussion* section.

In Fig. 4, results from Specimen 3 with high hyperplasia are presented. Benign prostatic hyperplasia (BPH) is a process resulting in the expansion of glandular-epithelial and stromal tissue, as well as muscle tissue leading to an enlarged prostate [43]. In Specimen 3, hyperplastic changes resulted in the slice being larger than the imaging FOV and was therefore bisected. Here, we present the left part, where most of the tumor was located. This specimen was further divided into two sections during histology preparation, as in Fig. 4(a). The left section contains a central region with an advanced tumor, which can be seen more clearly in the magnified image in Fig. 4(e). Peripheral zone glandular areas located above and below the region of cancer are structured in patterns specific to BPH. Along the left edge of this section, residual prostate capsule and periprostatic tissue are visible. The right-hand side of specimen in Fig. 4(a) features larger glands characteristic of BPH and an extensive region of stroma. A portion of this area in the yellow square is magnified in Fig. 4(i).

The OCT image of Specimen 3 is presented in Fig. 4(b). Interestingly, in this case, the OCT intensity is not uniform and shows high variability with the notably brighter region in the bottom-left part of this image partially covering the co-registered region of the tumor, as presented in the magnification in Fig. 4(f). Stromal fibrous structures are also well contrasted and visible as bands of high intensity. In Fig. 4(j), a magnified OCT image presenting the region indicated by the yellow square in Fig. 4(b) shows the glands visible in the area of uninvolved tissue. These glands remain mostly filled with prostate fluid, as confirmed by the histology image in Fig. 4(i) (yellow arrows). As suggested in [22], this may contribute to the relatively low contrast visible between glands and surrounding stroma in Fig. 4(j).

In the strain image (Fig. 4(c)), in the left section of the specimen there is a distinctive region of low, relatively homogeneous absolute strain (∼0 mε) that spans over the region containing tumor in the corresponding histology. This region is magnified in Fig. 4(g). Interestingly, the region of homogeneous strain extends into the region corresponding to no tumor in histology. As with Specimen 2, it may be caused by differences in the geometry of histology and OCE scans or it might be because of some non-tumor regions showing similar patterns in OCE images. The right part of the specimen consists of normal tissue, mostly healthy glands, and is characterized by a highly variable strain extending from –1 mε to 1 mε, as shown in Fig. 4(k). In this projection, particular structures, like the glands indicated by the yellow arrows, can hardly be differentiated.

The elasticity image is shown in Fig. 4(d). Almost the entire left section of the image shows high elasticity (>100 kPa), particularly in the area containing tumor, magnified in Fig. 4(h). The left boundary of this section, corresponding to the periprostatic tissue, shows lower elasticity, comparable to the region of healthy stroma. It is noteworthy that the area of increased elasticity in the left section is much larger than the area of cancer in the histology image. A possible explanation for this is an increase in elasticity due to BPH changes in the tissue adjacent to the tumor. Another explanation could be the imperfect matching of the histology and elasticity imaging planes. The region of healthy tissue, featuring low average elasticity (∼10 kPa) and a high percentage of gaps with missing elasticity due to positive strain, is presented in Fig. 4(l). Here, the locations with bigger glands identified in Fig. 4(j) are marked with yellow arrows and present as regions of increased elasticity, which is common in case of changes caused by BPH.

Figure 5 shows an example of a sample featuring extensive regions of normal glands with tumor also present (Specimen 4). The histology image in Fig. 5(a) presents areas with large numbers and various sizes of glands in the upper and bottom-right regions of the image, characteristic of a peripheral zone of a prostate. A magnification portion of this area indicated with the yellow square is shown in Fig. 5(i), revealing complex shapes of the individual glands. The inner wall exhibits a strongly differentiated structure, characterized by numerous branched protrusions specific to healthy glands. The lower-left part of the specimen is covered by regions of tumor (mostly Gleason grade 4). A magnification of the selected region, marked with a green square in Fig. 5(a), is shown in Fig. 5(e). In this area, except for the very upper part, where normal glands are present, the tissue exhibits a compact structure formed by poorly formed glands with largely compressed lumens. It is also well-stained due to the increased density of cell nuclei. Only a small number of distinguishable glands are visible in the affected area.

Figure 5(b) shows the OCT image obtained at a depth of ∼80 µm below the tissue surface. The regions in square boxes correspond to co-registered areas of normal glands and tumor. Magnified images of these areas are shown in Fig. 5(j) and Fig. 5(f), respectively. The tumor area presents with lighter bands of OCT intensity, which correspond to fibromuscular structures. In contrast, the healthy area shows a high degree of differentiation, with both empty and fluid-filled prostate glands. Both areas exhibit similar OCT intensity levels.

Figures 5(c) and 5(d) show strain and elasticity images, respectively. Enlarged areas marked with squares are presented in Fig. 5(k) and Fig. 5(l) for the uninvolved tissue and Fig. 5(g) and Fig. 5(h) for the tumor, respectively. In the case of the tumor, uniform near-zero strain correlated with elevated elasticity (>100 kPa) is observed. This is particularly evident in the lower-right part of the images. Figure 5(g) and Fig. 5(h) provide better contrast of the structures that are difficult to distinguish in the corresponding OCT image (Fig. 5(f)). The upper part of this area shows greater strain variation and lower elasticity (<10 kPa). This indicates the different mechanical characteristics between the two parts. The presence of cancerous tissue is confined only to the lower area with higher elasticity, and the border between the cancerous and normal tissue is clearly visible. Figure 5(k) shows the healthy area, where the strain image is highly varied due to the randomized behavior of the glands. A large percentage of the area exhibits positive strain, resulting in undefined elasticity, as seen in Fig. 5(l). In locations where elasticity can be correctly calculated, it exhibits low values, as expected.

Figure 6 shows a prostate specimen (Specimen 5) with tumor regions of varying Gleason grades (from 3 to 5). The dominant lesion can be observed in the histology specimen in Fig. 6(a) as an extensive, well-stained area on the right-hand side. Within this area, individual structures can be distinguished, forming characteristic regions with a Gleason grade ranging from 3 to 5. A selected region from this area is marked with a green square, and its magnification is shown in Fig. 6(e). The upper and lower sections of the slice contain areas of clearly visible and well-formed healthy glands of dense distribution. The selected upper region is marked with a yellow square and its magnified projection is presented in Fig. 6(i).

Figure 6(b) shows an OCT image obtained at a depth of ∼60 µm below the tissue surface. Figure 6(c) shows the corresponding strain image, and Fig. 6(d) presents the elasticity image. The parts of the OCT images corresponding to the sections of the healthy glands and tumor areas are marked with yellow (region of healthy glands) and green (tumor) squares. The magnified images of these areas are shown in Fig. 6(j) for the normal glands and in Fig. 6(f) for the tumor. The cancerous region in the OCT image is characterized by a low level of intensity variation. Only a few empty glands embedded in a homogeneous structure formed by merged glands along with distinct bands of fibromuscular stroma structures can be identified. In contrast, areas of healthy glands reveal many glands of varying sizes, with well-contrasted bright boundaries.

The strain and elasticity images reinforce previously noted characteristics of healthy versus tumor-affected tissue. The magnified region containing the tumor (Fig. 6(g)) shows large areas of low strain. Only the top-right corner of this image, where a fragment of healthy tissue is located, shows increased heterogeneity. Similarly, in Fig. 6(k), which shows a region with healthy glands, the high variability of strain provides additional contrast of structures, such as prostate glands. However, due to the largely randomized behavior of glands under stress, it is often challenging to identify the shapes of individual glands in the strain image. Therefore, the strain image in Fig. 6(k) should be interpreted holistically as presenting an area of complexity formed by structures prone to non-uniform deformation. In contrast, the tumor-affected area in the elasticity image (Fig. 6(h)) is characterized by a smooth gradient in elasticity between the specific structures. The average elasticity of this region is higher than the corresponding value for the benign tissue (Fig. 6(l)), as expected.

## 4. Discussion

Elastography provides an opportunity to diagnose prostate cancer using stiffness as a biomarker. Previous studies have demonstrated the potential of OCE in identifying prostate cancer in biopsy samples [31,32]. However, these studies were limited to small, fixed samples of the prostate. In our approach, we focused on the examination of fresh prostate samples, providing conditions more closely matched to the clinical scenario. We utilized a mosaicking protocol with a total lateral FOV of 46 × 46 mm^2^, providing images of whole prostate cross-sections. Using QME, we generated OCT, strain, and elasticity images. An important component of our study was the validation of our results through co-registration with gold-standard histology.

**Fig. 7.**
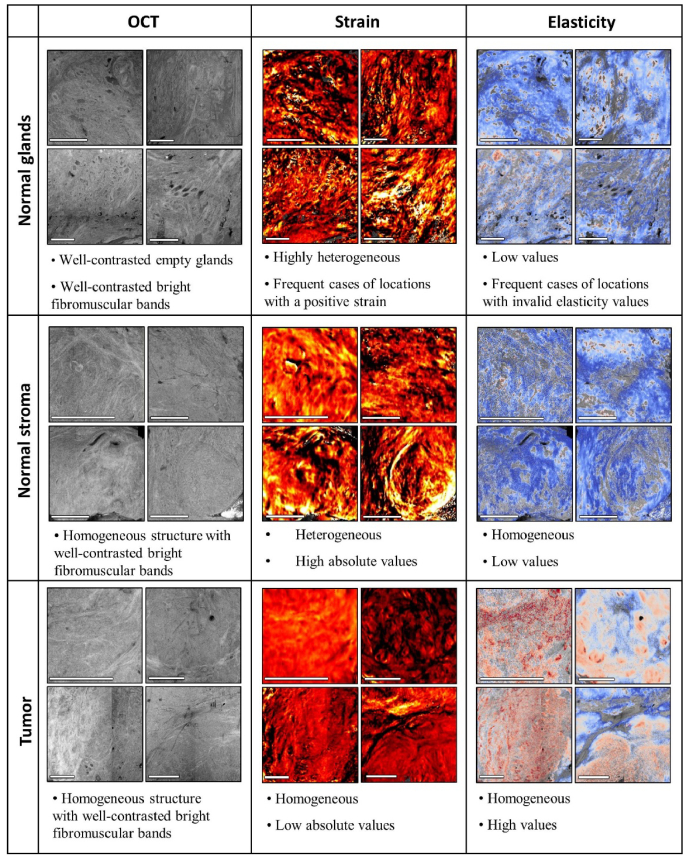
Summary of the findings in QME imaging of the freshly excised prostate presented in the manuscript in qualitative terms. The characteristics of the OCT, strain and elasticity images are illustrated by exemplary images of normal glands, normal stroma and tumor. Scale bar: 2 mm.

The strain and elasticity images obtained provide a contrast of prostate structures that is complementary to OCT images. In particular, QME enhances the differentiation of tumor, which is often stiffer than benign tissue. Whereas tumor (Gleason grade ≥ 4) in OCT images presents as a relatively uniform structure, interlaced with distinguishable bands of stroma, it often does not differ from the presentation of benign tissue and, therefore, cannot be differentiated reliably. On the contrary, in strain images, the dense, compact cancerous tissue often presents as homogeneous regions of low absolute strain. This corresponds to uniform areas of high elasticity. On the other hand, the complex structure of normal fibromuscular stroma provides high heterogeneity in strain images. The features visible in strain images, in some cases, correspond to particular structures visible in co-registered histology, whereas in others, they show no obvious correlation with histology images. This can be the effect of non-uniform stress, which can occur, for example, in the case of a particularly rough sample surface, resulting in inaccurate adhesion of the compliant silicone layer. In contrast, the non-uniformity of the stress is partially compensated in the calculation of the local elasticity, where such regions are represented by uniformly low elasticity, as expected. The key findings of the study are summarized in Fig. 7.

In the descriptions of our results, we referred to increased and decreased elasticity values relative to the mean value for the specimen. Higher values of elasticity, usually defined as higher than 100 kPa, according to the co-registration results, correspond to tumor, whereas values lower than 10 kPa correspond to regions of benign tissue. Therefore, we propose the intermediate value of 50 kPa as the threshold allowing classification of particular areas as cancerous regions. This value corresponds well to the findings in ultrasound elastography [44,45]. Ultrasound shear-wave elastography (USWE) enables the elastic modulus to be obtained by directly measuring the induced shear-wave velocity and assuming known sample density. The technique was shown to provide high specificity and sensitivity in clinically significant prostate cancer detection [46]. The threshold for elastic modulus in the prostate measured using USWE is typically determined to be in the range of 35-85 kPa, and the spread of these results depends on the patient cohort included in the study and the defined confidence interval [44,45]. Further assignment of the average elastic modulus ranges of the whole prostate to the Gleason score using USWE has also been proposed [47]. Our approach does not distinguish between Gleason grades 3, 4, and 5 at this stage. However, the feasibility of such a distinction by a higher resolution OCT system and statistical and correlation analysis performed on a larger collection of specimens is planned in a future study.

Our findings in this pilot study demonstrate that QME provides contrast to differentiate tumor from benign tissue. The next step towards the clinical application of QME in prostate cancer treatment is a larger *ex vivo* study to establish the diagnostic accuracy of the method by evaluating its sensitivity and specificity. Separately, the application of QME in prostate cancer requires *in vivo* application, both intraoperatively and for biopsy guidance. As described in the Introduction section, to overcome the problem of limited penetration depth of OCT/OCE (∼1 mm in dense tissue), the scanning head can be adapted to the form factor of a needle-like endoscope. A related proof-of-principle OCE needle probe has been demonstrated in breast tissue [48,49]. Extending this device to perform needle-based QME could provide support in transurethral resection of the prostate.

The approach applied in our study, despite its potential in detecting tumor, has some limitations that may influence its accuracy. One issue is the protocol used for co-registration with histology. The same surface of the sample that was scanned with OCE also served as the slicing interface in the microdissection step of the histology slide preparation process. Only the interface from a certain depth below the surface of the specimen that meets the quality requirements undergoes slide preparation and scanning using the histology slide scanner. The 3-D OCE data allows for determining the depth in tissue at which the co-registration has the best match. However, several aspects make this procedure challenging. Firstly, there is a difference in the scale of the images. In the H&E staining procedure, the specimen can shrink due to dehydration, clearing, and embedding in paraffin wax up to 20% of the original size of the fresh specimen [50]. In addition, the lateral extension of the slice and its components in the OCT image is generally increased by the pre-load stress applied to the sample before scanning. The level of stress required depends on the sample's average elasticity and surface roughness. Secondly, the angle of the cut made by the microtome does not necessarily match the angle of the OCE *en face* projection. Consequently, only a limited portion of the two images may show accurate correspondence. Nevertheless, in most cases, it is possible to proceed with the level of similarity obtained and use them for the interpretation of OCT images and elastograms. A possible approach to improve the co-registration could be the use of a more advanced protocol for the manipulation of the 3-D OCT and QME data, enabling sectioning at any angle to improve matching with histology [51].

Another challenge is the occurrence of positive strain under uniaxial load. As compression is applied across the entire sample surface, factors such as friction at the sample boundaries, uneven surface topology, local mechanical heterogeneity, and incompressibility may lead to inaccuracies in the measured strain. In some cases, positive strain is obtained, which corresponds to axial elongation of the tissue under compressive stress. This is most likely due to mechanical interaction with the adjacent tissue. An example of such local regions of mechanical heterogeneity and uneven tissue surface includes groups of larger glands embedded within the normal stroma in the prostate. In our results, these regions are represented by segments showing high strain variability. As elasticity, by definition, is positive, locations with positive strain and negative stress are removed from the images to avoid confounding the subsequent analysis. To address this issue, a refined approach based on the measurement of the 3-D displacement vector field can be used [41]. In this method, the full strain tensor is calculated from the amplitude of the complex correlation coefficients across multiple digitally shifted images. Access to the strain tensor components enables the use of a number of measures providing mechanically meaningful contrast in the locations where uniaxial analysis breaks.

## 5. Conclusions

In this study, we demonstrated 3-D imaging of fresh prostate specimens with OCT, strain, and elasticity. Wide-field scanning mode enabled imaging of the entire prostate cross-sections improving accuracy of the co-registration of *en face* projections with histology. The images provide localized information on the mechanical properties of both benign and cancerous tissue. We characterized the strain and elasticity images in healthy and cancerous tissue indicating differences between them enabling, in most cases, differentiation between the two types of tissue. The results presented provide positive preliminary evidence of the potential of QME to effectively support oncology procedures such as imaged-guided biopsy by using a needle-like OCE probe design. The plan for further work provides for the collection of measurement data in sufficient quantity to enable a reliable quantitative description of the observations made in regard to clinical accuracy.

## Data Availability

Data underlying the results presented in this paper are not publicly available at this time but may be obtained from the authors upon reasonable request.

## References

[1] W. H. O. International Agency for Research on Cancer, (2022), retrieved https://gco.iarc.who.int/en.

[2] Corsini C. Bergengren O. Carlsson S. et al., “Patient-reported Side Effects 1 Year After Radical Prostatectomy or Radiotherapy for Prostate Cancer: A Register-based Nationwide Study,” European Urology Oncology 7(3), 605–613 (2024). 10.1016/j.euo.2023.12.007 38233329 PMC11102330

[3] Serefoglu E. C. Altinova S. Ugras N. S. et al., “How reliable is 12-core prostate biopsy procedure in the detection of prostate cancer?” Can. Urol. Assoc. J. 7(5-6), 293–298 (2013). 10.5489/cuaj.1248 PMC3668408 22398204

[4] Dias A. B. Ghai S., “Prostate Cancer Diagnosis with Micro-ultrasound: What We Know now and New Horizons,” Radiologic Clinics of North America 62(1), 189–197 (2024). 10.1016/j.rcl.2023.06.014 37973243

[5] Napoli A. Giulia A. Roberto S. et al., “High-intensity focused ultrasound for prostate cancer,” Expert Rev. Med. Devices 17(5), 427–433 (2020). 10.1080/17434440.2020.1755258 32275187

[6] Fernandes M. C. Yildirim O. Woo S. et al., “The role of MRI in prostate cancer: current and future directions,” Magn. Reson. Mater. Phy. 35(4), 503–521 (2022). 10.1007/s10334-022-01006-6 PMC9378354 35294642

[7] Das C. J. Razik A. Netaji A. et al., “Prostate MRI-TRUS fusion biopsy: a review of the state of the art procedure,” Abdom. Radiol. 45(7), 2176–2183 (2020). 10.1007/s00261-019-02391-8 31897683

[8] Correia E. T. d. O. Baydoun A. Li Q. et al., “Emerging and anticipated innovations in prostate cancer MRI and their impact on patient care,” Abdominal Radiology 49(10), 3696–3710 (2024). 10.1007/s00261-024-04423-4 38877356 PMC11390809

[9] Chatterjee A. Mercado C. Bourne R. M. et al., “Validation of Prostate Tissue Composition by Using Hybrid Multidimensional MRI: Correlation with Histologic Findings,” Radiology 302(2), 368–377 (2022). 10.1148/radiol.2021204459 34751615 PMC8805656

[10] Lo W.-C. Bittencourt L. K. Panda A. et al., “Multicenter Repeatability and Reproducibility of MR Fingerprinting in Phantoms and in Prostatic Tissue,” Magn. Reson. Med. 88(4), 1818–1827 (2022). 10.1002/mrm.29264 35713379 PMC9469467

[11] Brunsing R. L. Schenker-Ahmed N. M. White N. S. et al., “Restriction spectrum imaging: An evolving imaging biomarker in prostate MRI,” J. Magn. Reson. Imaging 45(2), 323–336 (2017). 10.1002/jmri.25419 27527500 PMC5222783

[12] Tajaldeen A. Alrashidi M. Alsaadi M. J. et al., “Photoacoustic imaging in prostate cancer: A new paradigm for diagnosis and management,” Photodiagnosis Photodyn. Ther. 47, 104225 (2024). 10.1016/j.pdpdt.2024.104225 38821240

[13] Gomes E. F. A. Paulino Junior E. de Lima M. F. R. et al., “Prostate cancer tissue classification by multiphoton imaging, automated image analysis and machine learning,” J. Biophotonics 16(6), e202200382 (2023). 10.1002/jbio.202200382 36806587

[14] Gaba F. Tipping W. J. Salji M. et al., “Raman Spectroscopy in Prostate Cancer: Techniques, Applications and Advancements,” Cancers 14(6), 1535 (2022). 10.3390/cancers14061535 35326686 PMC8946151

[15] Schueth A. Hildebrand S. Samarska I. et al., “Efficient 3D light-sheet imaging of very large-scale optically cleared human brain and prostate tissue samples,” Commun. Biol. 6(1), 170 (2023). 10.1038/s42003-023-04536-4 36781939 PMC9925784

[16] Drexler W. Fujimoto J. G., *Optical Coherence Tomography Technology and Applications* , 2nd ed. (Springer International Publishing, 2015).

[17] D’Amico A. V. Weinstein M. Li X. et al., “Optical coherence tomography as a method for identifying benign and malignant microscopic structures in the prostate gland,” Urology 55(5), 783–787 (2000). 10.1016/S0090-4295(00)00475-1 10792101

[18] Dangle P. P. Shah K. K. Kaffenberger B. et al., “The use of high resolution optical coherence tomography to evaluate robotic radical prostatectomy specimens,” Int. Braz. J. Urol. 35(3), 344–353 (2009). 10.1590/S1677-55382009000300011 19538770

[19] Gardecki J. A. Singh K. Wu C. L. et al., “Imaging the Human Prostate Gland Using 1-mum-Resolution Optical Coherence Tomography,” Arch. Pathol. Lab Med. 143(3), 314–318 (2019). 10.5858/arpa.2018-0135-OA 30550349

[20] Muller B. G. de Bruin D. M. van den Bos W. et al., “Prostate cancer diagnosis: the feasibility of needle-based optical coherence tomography,” J. Med. Imag. 2(3), 037501 (2015). 10.1117/1.JMI.2.3.037501 PMC4498002 26171414

[21] Swaan A. Mannaerts C. K. Muller B. G. et al., “The First In Vivo Needle-Based Optical Coherence Tomography in Human Prostate: A Safety and Feasibility Study,” Lasers Surg. Med. 51(5), 390–398 (2019). 10.1002/lsm.23093 31090088 PMC6617991

[22] Skrok M. K. Tamborski S. Hepburn M. S. et al., “Imaging of prostate micro-architecture using three-dimensional wide-field optical coherence tomography,” Biomed. Opt. Express 15(12), 6816 (2024). 10.1364/BOE.537783 39679405 PMC11640564

[23] Swaan A. Muller B. G. Wilk L. S. et al., “One-to-one registration of en-face optical coherence tomography attenuation coefficients with histology of a prostatectomy specimen,” J. Biophotonics 12(4), e201800274 (2019). 10.1002/jbio.201800274 30565879

[24] Szymańska K. Hainaut P., “Prostate Cancer: Diagnosis and Treatment,” in *Encyclopedia of Cancer (Third Edition)* , Boffetta P. Hainaut P., eds. (Academic Press: Oxford, 2019), pp. 292–298.

[25] Almalki Y. E. Mansour M. G. E.-D. Ali S. A. et al., “Advanced strain elastography is a reliable approach for prostate cancer detection in patients with elevated PSA levels,” Sci. Rep. 14(1), 2917 (2024). 10.1038/s41598-024-53440-2 38316992 PMC10844258

[26] Kennedy B. F. Wijesinghe P. Sampson D. D., “The emergence of optical elastography in biomedicine,” Nat. Photonics 11(4), 215–221 (2017). 10.1038/nphoton.2017.6

[27] Larin K. V. Sampson D. D., “Optical coherence elastography - OCT at work in tissue biomechanics [Invited],” Biomed. Opt. Express 8(2), 1172–1202 (2017). 10.1364/BOE.8.001172 28271011 PMC5330567

[28] Gubarkova E. V. Kiseleva E. B. Sirotkina M. A. et al., “Diagnostic Accuracy of Cross-Polarization OCT and OCT-Elastography for Differentiation of Breast Cancer Subtypes: Comparative Study,” Diagnostics 10(12), 994 (2020). 10.3390/diagnostics10120994 33255263 PMC7760404

[29] Kennedy K. M. Zilkens R. Allen W. M. et al., “Diagnostic Accuracy of Quantitative Micro-Elastography for Margin Assessment in Breast-Conserving Surgery,” Cancer Res. 80(8), 1773–1783 (2020). 10.1158/0008-5472.CAN-19-1240 32295783

[30] Gong P. Chin S. L. Allen W. M. et al., “Quantitative Micro-Elastography Enables In Vivo Detection of Residual Cancer in the Surgical Cavity during Breast-Conserving Surgery,” Cancer Res. 82(21), 4093–4104 (2022). 10.1158/0008-5472.CAN-22-0578 36098983 PMC9627129

[31] Li C. Guan G. Ling Y. et al., “Detection and characterisation of biopsy tissue using quantitative optical coherence elastography (OCE) in men with suspected prostate cancer,” Cancer Lett. 357(1), 121–128 (2015). 10.1016/j.canlet.2014.11.021 25444932

[32] Yuting L. Li C. Zhou K. et al., “Microscale characterization of prostate biopsies tissues using optical coherence elastography and second harmonic generation imaging,” Lab Invest. 98(3), 380–390 (2018). 10.1038/labinvest.2017.132 29251735 PMC5842892

[33] Ling Y. Li C. Feng K. et al., “Effects of fixation and preservation on tissue elastic properties measured by quantitative optical coherence elastography (OCE),” J. Biomech. 49(7), 1009–1015 (2016). 10.1016/j.jbiomech.2016.02.013 26903410

[34] Kennedy K. M. Chin L. McLaughlin R. A. et al., “Quantitative micro-elastography: imaging of tissue elasticity using compression optical coherence elastography,” Sci. Rep. 5(1), 15538 (2015). 10.1038/srep15538 26503225 PMC4622092

[35] Allen W. M. Kennedy K. M. Fang Q. et al., “Wide-field quantitative micro-elastography of human breast tissue,” Biomed. Opt. Express 9(3), 1082–1096 (2018). 10.1364/BOE.9.001082 29541505 PMC5846515

[36] Li J. Pijewska E. Fang Q. et al., “Analysis of strain estimation methods in phase-sensitive compression optical coherence elastography,” Biomed. Opt. Express 13(4), 2224–2246 (2022). 10.1364/BOE.447340 35519281 PMC9045929

[37] Metzner K. L. Fang Q. Sanderson R. W. et al., “Analysis of friction in quantitative micro-elastography,” Biomed. Opt. Express 14(10), 5127–5147 (2023). 10.1364/BOE.494013 37854567 PMC10581800

[38] Epstein J. I., “Prostate cancer grading: a decade after the 2005 modified system,” Mod. Pathol. 31, 47–63 (2018). 10.1038/modpathol.2017.133 29297487

[39] Woo S. Kim S. Y. Cho J. Y. et al., “Shear wave elastography for detection of prostate cancer: a preliminary study,” Korean J. Radiol. 15(3), 346–355 (2014). 10.3348/kjr.2014.15.3.346 24843239 PMC4023053

[40] Kennedy B. F. McLaughlin R. A. Kennedy K. M. et al., “Investigation of Optical Coherence Microelastography as a Method to Visualize Cancers in Human Breast Tissue,” Cancer Res. 75(16), 3236–3245 (2015). 10.1158/0008-5472.CAN-14-3694 26122840

[41] Wijesinghe P. Chin L. Kennedy B. F., “Strain Tensor Imaging in Compression Optical Coherence Elastography,” IEEE J. Sel. Top. Quantum Electron. 25(1), 1–12 (2019). 10.1109/JSTQE.2018.2871596

[42] Tyekucheva S. Bowden M. Bango C. et al., “Stromal and epithelial transcriptional map of initiation progression and metastatic potential of human prostate cancer,” Nat. Commun. 8(1), 420 (2017). 10.1038/s41467-017-00460-4 28871082 PMC5583238

[43] Roehrborn C. G., “Pathology of benign prostatic hyperplasia,” Int. J. Impot. Res. 20(S3), S11–S18 (2008). 10.1038/ijir.2008.55 19002119

[44] Boehm K. Salomon G. Beyer B. et al., “Shear wave elastography for localization of prostate cancer lesions and assessment of elasticity thresholds: implications for targeted biopsies and active surveillance protocols,” J. Urol. 193(3), 794–800 (2015). 10.1016/j.juro.2014.09.100 25264337

[45] Dai W.-B. Xu J. Yu B. et al., “Correlation of Stiffness of Prostate Cancer Measured by Shear Wave Elastography with Grade Group: A Preliminary Study,” Ultrasound in Medicine & Biology 47(2), 288–295 (2021). 10.1016/j.ultrasmedbio.2020.10.018 33234327

[46] Sang L. Wang X.-M. Xu D.-Y. et al., “Accuracy of shear wave elastography for the diagnosis of prostate cancer: A meta-analysis,” Sci. Rep. 7(1), 1949 (2017). 10.1038/s41598-017-02187-0 28512326 PMC5434001

[47] Ji Y. Ruan L. Ren W. et al., “Stiffness of prostate gland measured by transrectal real-time shear wave elastography for detection of prostate cancer: a feasibility study,” Br. J. Radiol. 92(1097), 20180970 (2009). 10.1259/bjr.20180970 PMC6580922 30875242

[48] Kennedy K. M. McLaughlin R. A. Kennedy B. F. et al., “Needle optical coherence elastography for the measurement of microscale mechanical contrast deep within human breast tissues,” J. Biomed. Opt. 18(12), 121510 (2013). 10.1117/1.JBO.18.12.121510 24365955

[49] Kennedy K. M. Kennedy B. F. McLaughlin R. A. et al., “Needle optical coherence elastography for tissue boundary detection,” Opt. Lett. 37(12), 2310–2312 (2012). 10.1364/OL.37.002310 22739891

[50] Winsor L., “Tissue Processing,” in *Laboratory histopathology: a complete reference* (Churchill Livingstone, 1994).

[51] Sanderson R. W. Zilkens R. Gong P. et al., “A co-registration method to validate in vivo optical coherence tomography in the breast surgical cavity,” Heliyon 11(1), e41265 (2025). 10.1016/j.heliyon.2024.e41265 39807517 PMC11728906

