# Wide-field quantitative micro-elastograph of freshly excised human prostate: publisher’s note

**DOI:** 10.1364/BOE.614205

**Published:** 2026-08-17

**Authors:** Szymon Tamborski, Marta K. Skrok, Matt S. Hepburn, Mateusz Maniewski, Marek Zdrenka, Adam Kowalewski, Łukasz Szylberg, Brendan F. Kennedy

**Affiliations:** 1 Institute of Physics, Faculty of Physics, Astronomy and Informatics, Nicolaus Copernicus University in Toruń, 5 Grudziądzka St., 87-100 Toruń, Poland; 2 Department of Electrical, Electronic and Computer Engineering, School of Engineering, The University of Western Australia, 35 Stirling Highway, Perth, 6009, Western Australia, Australia; 3 BRITElab, Harry Perkins Institute of Medical Research, QEII Medical Centre Nedlands and Centre for Medical Research, The University of Western Australia, Perth, Western Australia 6009, Australia; 4 Department of Obstetrics, Gynaecology and Oncology, Chair of Pathomorphology and Clinical Placentology, Collegium Medicum in Bydgoszcz, Nicolaus Copernicus University in Toruń, 75 Ujejskiego St., Bydgoszcz 85-168, Poland; 5 Department of Tumor Pathology and Pathomorphology, Oncology Centre Prof. Franciszek Łukaszczyk Memorial Hospital, 2 Romanowskiej St., Bydgoszcz 85-796, Poland; 6 Faculty of Medicine, Bydgoszcz University of Science and Technology, Prof. S. Kaliskiego Avenue 7, 85-796 Bydgoszcz, Poland

## Abstract

This publisher’s note contains a correction to the funding of [ Biomed. Opt. Express 16, 3027 (2025)10.1364/BOE.563310PMC1233930140809985]. The article was corrected on 10 August 2026.

In [1], the funding section was amended to include the award number.
